# Tuning the functionalities of a mesocrystal via structural coupling

**DOI:** 10.1038/srep12073

**Published:** 2015-07-14

**Authors:** Heng-Jui Liu, Yun-Ya Liu, Chih-Ya Tsai, Sheng-Chieh Liao, Ying-Jiun Chen, Hong-Ji Lin, Chih-Huang Lai, Wen-Feng Hsieh, Jiang-Yu Li, Chien-Te Chen, Qing He, Ying-Hao Chu

**Affiliations:** 1 Department of Materials Science and Engineering, National Chiao Tung University, Hsinchu 30010, Taiwan; 2 Department of Physics, Durham University, Durham DH1 3LE, United Kingdom; 3 School of Materials Science and Engineering, Key Laboratory of Low Dimensional Materials and Application Technology of Ministry of Education, Xiangtan University, Hunan 411105, China; 4 Department of Photonics and Institute of Electro-Optical Engineering, National Chiao Tung University, Hsinchu 30010, Taiwan; 5 Department of Materials Science and Engineering, National Tsing Hua University, Hsinchu 30013, Taiwan; 6 National Synchrotron Radiation Research Center, Hsinchu 30076, Taiwan; 7 Department of Mechanical Engineering, University of Washington, Seattle, WA 98195–2600, USA; 8 Institute of Physics, Academia Sinica, Taipei 105, Taiwan

## Abstract

In the past decades, mesocrystal, a kind of nanocrystals with specific crystallographic orientation, has drawn a lot of attention due to its intriguing functionalities. While the research community keeps searching for new mesocrystal systems, it is equally crucial to develop new approaches to tune the properties of mesocrystals. In this work, a self-organized two-dimensional mesocrystal composed of highly oriented CoFe_2_O_4_ (CFO) nano-crystals with assistance of different perovskite matrices is studied as a model system. We have demonstrated that the strain state and corresponding magnetic properties of the CFO mesocrystal can be modulated by changing the surrounding perovskite matrix through their intimate structural coupling. Interestingly, this controllability is more strongly correlated to the competition of bonding strength between the matrices and the CFO mesocrystals rather than the lattice mismatch. When embedded in a matrix with a higher melting point or stiffness, the CFO mesocrystal experiences higher out-of-plane compressive strain and shows a stronger magnetic anisotropy as well as cation site-exchange. Our study suggests a new pathway to tailor the functionalities of mesocrystals.

A new class of crystals, mesocrystals, which is constructed by nanoscale building units, shows a perfect alignment in crystallographic orientation that can be found in single crystals. This kind of superstructures has stimulated wide interests in the research fields of condensed matter physics and solid state chemistry for their unique properties that are different from bulk single crystals and randomly assembled nanocrystals^1^,^2^,^3^,^4^. Current methods to prepare the mesocrystals are mostly based on the chemical synthesis, starting from colloidal nanoparticles and then naturally assembled by means of organic matrices, physical fields, mineral bridges, *etc*^2^. Recently, the definition of “mesocrystal” has been refined to refer to the feature of superstructures instead of the formation mechanism^5^. Based on this definition, the mesocrystal can be extended to cover those crystallographically oriented nanocrystals embedded in an organic or inorganic matrix.

The functionalities of mesocrystals are the collective behaviors of composed nanocrystals. Thus, tuning the intrinsic properties of the constituent nanocrystals is one of the essential factors to determine the properties of mesocrystals. However, matrix, the other important part of the composite system, has been only treated to assemble nanocrystals into an array with specific crystallographic orientation. Little attention has been paid to understand the influence of the matrix on the properties of these nanocrystals. In order to unveil this relationship between the matrix and the nanocrystals, self-assembled epitaxial nanocomposites synthesized by physical deposition process are used as an example. In these systems, two oxide materials, spinels and perovskites, are served as nanopillars and matrix, respectively. The spontaneously assembled nanopillars also possess perfectly ordered crystal-orientation, which can be viewed as a 2-dimenstional (2D) mesocrystal system^6^,^7^,^8^,^9^. The perovskite matrix can establish compact connection with the spinel mesocrystal through the bridge of octahedral sites^10^, which strongly affects the structural and physical properties of the mesocrystals^11^,^12^,^13^,^14^. Therefore, the matrix materials become very important since they provide additional degrees of freedom to tailor the crystal orientations and physical properties of the mesocrystal, which cannot be observed in those mesocrystals within the organic/polymer matrices.

In this work, spinel CoFe_2_O_4_ (CFO) and several perovskties (BiFeO_3_, PbTiO_3_, SrTiO_3_, and SrRuO_3_) have been chosen based on the following reasons. Firstly, while CFO and perovskite materials codeposit on the perovskite substrates, CFO naturally forms mesocrystal due to the large surface energy at spinel and perovskite interface^7^. Secondly, ferrimagnetic CFO with strong negative magnetostrictive effect^12^, presenting a strong magnetic anisotropy along the compressive direction, is very sensitive to structural changes. We have found that the CFO mesocrystal undergoes different out-of-plane (OOP) compressive strain states when embedded in several commonly adopted perovskite matrices. The variation of strain state in the CFO mesocrystal also changes its magnetic properties such as a magnetic anisotropy or the origin of magnetic moment in atomic scale. More interestingly, these variations have been discovered to be highly dependent on the bonding strength of the perovskite materials instead of the lattice mismatch between the matrices and the CFO mesocrystal. This study delivers a new concept to design the stress-mediated functionalities of the mesocrystals via the chosen matrix materials.

## Results

**Figure 1:**
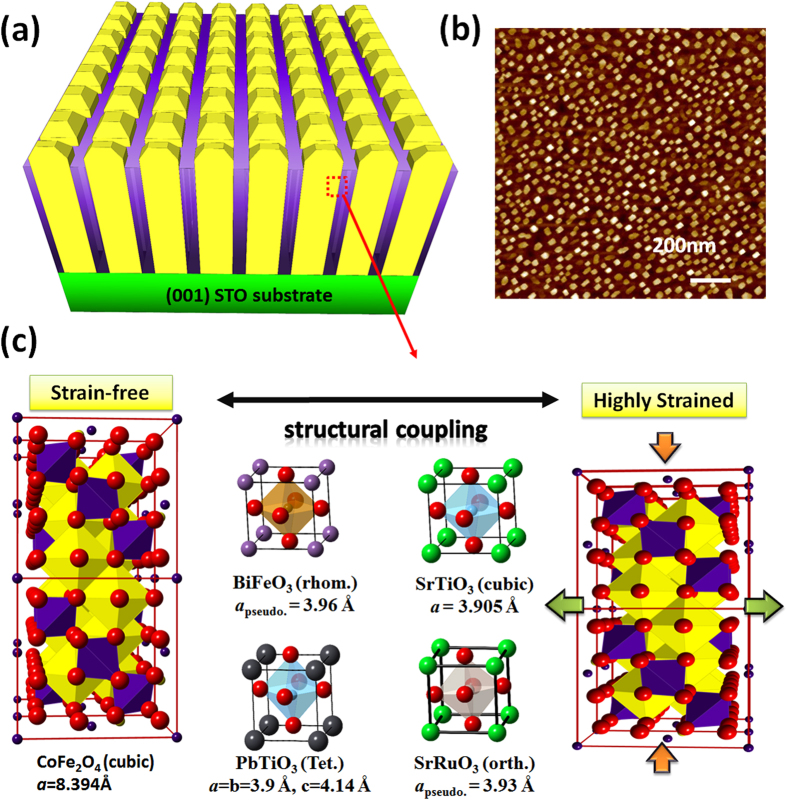
(**a**) The schematic illustration of a 2-D mesocrystal made from self-organized spinel CFO single-crystal nanocolumns. (**b**) The top view atomic force image (AFM) of this mesocrystal. (**c**) The scheme of modulating the strain state of the mesocrystal by varying the perovskite matrix.

Figure 1(a) illustrates the typical feature of a mesocrystal consisting of spinel CFO nanocrystals and a perovskite matrix grown on (001) oriented STO substrate^12^,^13^,^14^. Usually, the spinels and perovskites are almost immiscible from energetic consideration so that these two phases can be spontaneously separated while they are codeposited^7^. CFO has much less wetting ability on (001) oriented STO substrate than perovskite materials so that it prefers to form the mesocrystal, whereas perovskites become matrices (Fig. 1(b))^7^,^13^,^15^. In this case, the STO substrate only provides the control of crystallographic orientation without obvious clamping effect on the mesocrystal, and thus the observed variation in the lattice and magnetic properties of the mesocrystal would principally result from the interaction with matrix materials. Although CFO can always self-organize as a mesocrystal in these perovskite matrices, hitherto no work has discussed how to alter the properties of the CFO mesocrystal by changing the surrounding matrices. In order to realize this idea, several perovskite matrices with different lattice parameters such as BiFeO_3_ (BFO) (*c* ≈ 3.96 Å), PbTiO_3_ (PTO) (c ≈ 4.14 Å), SrTiO_3_ (STO) (*c* ≈ 3.90 Å), and SrRuO_3_ (SRO) (*c* ≈ 3.93 Å) have been adopted. Spinel CFO has a cubic structure with *a* = 8.394 Å. Hence, the connection form is that one unit cell of CFO conjugates with two unit cells of perovskites, and the lattice mismatch of CFO can then be extracted as 5.6%, 1.4%, 7.0%, and 6.3% for BFO, PTO, STO, and SRO matrices, respectively (Fig. 1(c)).

**Figure 2:**
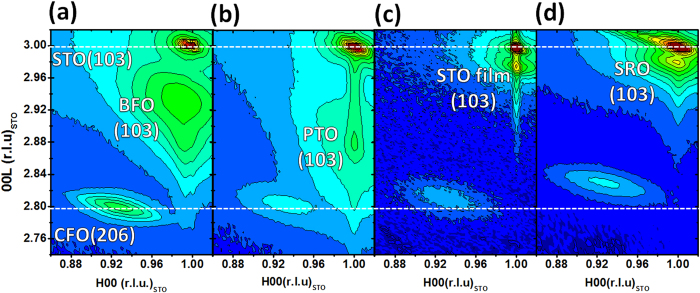
The set of X-ray reciprocal space maps shows that the (206) peak of the CFO mesocrystal moves toward high Miller index gradually while changing the conjugating matrix from (**a**) BFO, (**b**) PTO, (**c**) STO, and (**d**) SRO in sequence. This result confirms that the matrix can play an important role to manipulate the strain state of the CFO mesocrystal.

Based on the experiences learned from epitaxial systems, we would expect the strain state of the CFO mesocrystal to be strongly correlated to the lattice mismatch with perovskite matrices if they can coherently connect each other. However, to maintain the epitaxy between the CFO and various perovskite matrix materials, semi-coherent interfaces are inevitably originated to release strain by introducing defects such as dislocations, or oxygen vacancies^10^. Surprisingly, in these cases, even though one can find similar lattice mismatch between the mesocrystal and the surrounding matrix (for example, BFO, STO and SRO), the strain state of CFO are very different. To further realize such phenomenon, X-ray reciprocal space maps (RSMs) around the (103) diffraction of STO substrate were performed first to extract the structural information with respect to various perovskite matrices, mesocrystals, and the substrate as shown in Fig. 2. Two extra and clear diffraction spots near to the (103) plane of STO can be identified as (103) plane of perovskite matrices and (206) plane of the CFO mesocrystals, implying all systems have spontaneous phase separation and these constituents follow the cubic-on-cubic growth with STO substrate. In addition, two white dash lines are marked across these RSMs at L = 3.000 (*a*_STO_ = 3.905 Å) and L = 2.791 (*a*_CFO_ = 8.390 Å), which can be referred to the position of the c-axis lattice of single crystal substrate STO and bulk CFO. In these RSMs, the CFO (206) peak moves toward higher L index from the reference line of bulk CFO, which clearly exhibits an increasing compressive OOP strain of mesocrystals in the matrices of BFO, PTO, STO, and SRO in sequence. By carefully calculating the peak positions, the corresponding OOP lattice constants are extracted as 8.382 Å, 8.370 Å, 8.327 Å, and 8.284 Å, whereas the corresponding IP lattice constants are as 8.394 Å, 8.396 Å, 8.413 Å, and 8.443 Å. The corresponding OOP compressive/IP tensile strains of CFO can then be obtained as −0.1%/0.05%, −0.24%/0.1%, −0.67%/0.41%, and −1.2%/1%, using common strain equation (*ε* = *a/a*_0_−1, where *a* is the varied lattice constant and *a*_0_ is the bulk lattice constant). It is also noteworthy that the OOP and IP strains of these mesocrystals seem not to obey the correlation from the general Poisson equation of a film under biaxial strain (*ν* = 1/(1 − 2*ε*_*xx*_/*ε*_*zz*_)) with a prescribed Poisson’s ratio for standard CFO (*ν* ≈ 0.35). This implies that the CFO mesocrystals should have certain lattice defects such as oxygen vacancies, distortion or stoichiometric variation driven by surrounding matrices during the deposition, which make it difficult to identify the original lattice of CFO for each case and fail to apply the Poisson equation. Therefore, to express more clearly the stain-dependent trend for all samples, the OOP and IP strains from the common strain equation are adopted in this work. Further, compared to the lattice mismatch between the mesocrystals and matrices, no direct correlation can be addressed.

**Figure 3:**
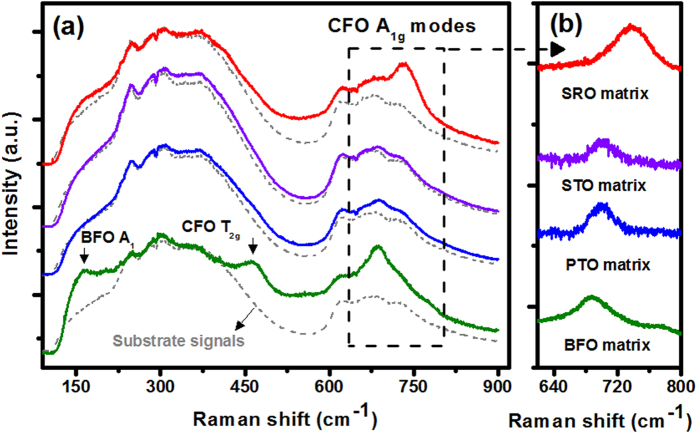
(**a**) The Raman spectra obtained from the CFO mesocrystal embedded in BFO (green line), PTO (blue line), STO (purple line), and SRO (red line) matrices. (**b**) The focused area of CFO A_1g_ phonon mode after carefully eliminating the background signal from STO substrate. The blue shift of the CFO A_1g_ peak also indicates the decrease of the CFO OOP lattice constant, consistent with the results of RSMs.

More evidence can be provided from Raman spectroscopy experiments shown in Fig. 3. Although a strong background signal from STO substrates overwhelms most of the phonon modes^16^,^17^,^18^, the Raman peaks from BFO and CFO can still be clearly detected owing to their smaller band gaps of 2.82 eV^19^,^20^ and 2.51 eV^21^, respectively. According to the rhombohedral *R*3*c* symmetry of BFO, the fingerprint around 162 cm^−1^, is assigned as A_1_ symmetry mode^19^,^22^. The peaks at around 700 cm^−1^ can then be identified as the A_1g_ phonon mode of the 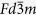
19,21,23 cubic spinel CFO. This phonon mode corresponds to the vibration of oxygen towards tetrahedral Fe, which can be utilized to characterize the lattice strains of CFO^18^,^19^,^23^,^24^. Comparing the phonon frequency of all samples as shown in Fig. 3(b), the A_1 g_ phonon softens with BFO matrix, slightly up shifts with PTO and STO matrices, and hardens significantly in SRO matrix. The hardened phonon frequency almost reveals a tetragonal-like symmetry^24^ and the blue shift of CFO A_1g_ mode confirms a compressive strain along OOP direction, consisting with the XRD results.

**Figure 4:**
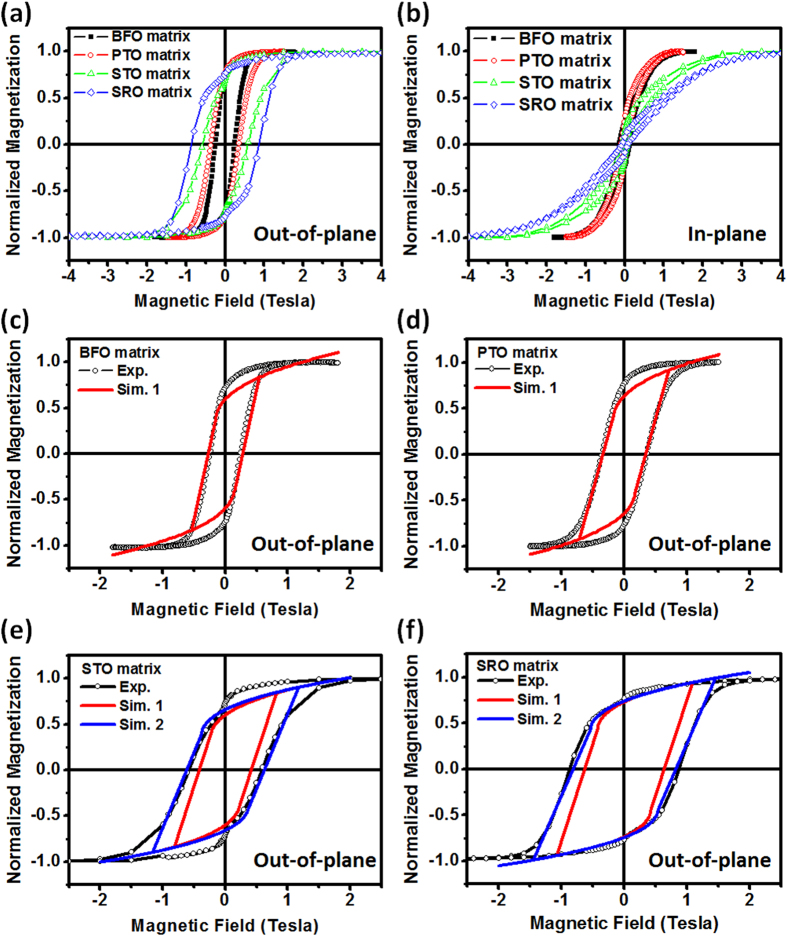
The hysteresis loops measured by applying the magnetic field along (**a**) out-of-plane (OOP) and (**b**) in-plane (IP) directions. (**c**–**f**) are the OOP hysteresis loops of BFO, PTO, STO, and SRO matrices simulated by a phenomenological thermodynamic theory, where Sim. 1 and Sim. 2 represent the simulation curve using anisotropy constant *k*_12_ ~ 2.7*10^−17^ J/A^4^ and *k*_12_ ~ 8.5*10^−17^ J/A^4^, respectively. The well-matched simulation curves of each case imply that the anisotropy constant can be variable under different strain state.

It is reminiscent that the magnetic properties of CFO vary sensitively to its strain state. From the perspective of macroscopic magnetic behavior as shown in Fig. 4(a,b), squarer and more broadened shape is presented in the OOP hysteresis loops while varying the matrices in sequence of BFO, PTO, STO to SRO, whereas more tilting and narrower shape in the IP hysteresis loops. The shape variation of these loops suggests that an enhancement of magnetic anisotropy toward OOP direction, which can be directly related to the increase of compressive strain along c-axis of the mesocrystals, in agreement with the results of XRD and Raman spectra. A phenomenological thermodynamic theory is then adopted to simulate OOP magnetic hysteresis loops. For a CFO mesocrystal under strain and magnetic field boundary condition, its free energy can be written as^25^:


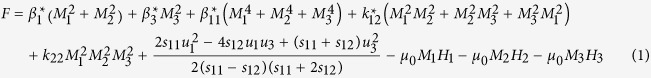


with


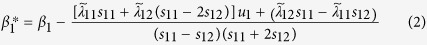



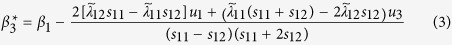



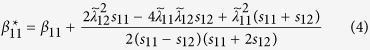



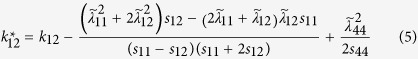


where *u*_1_ is in-plane strain, *u*_3_ is out-of-plane strain, *H*_i_ (*i* = 1, 2, 3) is magnetic field, *M*_i_ is magnetization, *β*_1_ and *β*_11_ are magnetic stiffness coefficients, *k*_12_ and *k*_22_ are the anisotropy constants, *s*_*IJ*_ (*I*, *J* = 1,2,. . .,6) is the elastic compliance at constant magnetization, 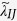 is the magnetostrictive coefficient, for which Voigt notation is used. The equilibrium magnetization under external magnetic field loading can be determined by minimization of the free energy. In this calculation, the anisotropy constant *k*_12_ is notably important because it directly associates with the shape of hysteresis loops. As shown in Fig. 4(c–f), the comparison between experimental (black lines) and simulated curves (red lines) shows a good consistency in the cases of small strained CFO mesocrystals (in BFO or PTO matrices) but a large difference in the cases of high strained ones (in STO or SRO matrices), while the anisotropy constant *k*_12_ is fixed at the value of 2.7*10^−17^ J/A^4^. This discrepancy in the high strained CFO mesocrystal can be further diminished after *k*_12_ is adjusted to 8.5*10^−17^ J/A^4^ as the blue lines in Fig. 4(e–f). Meanwhile, the calculated strain-induced magnetic anisotropy fields according to these well-matched simulated curves are around 7.5 kOe for BFO, 8.4 kOe for PTO, 29.9 kOe for STO, and 37.3 kOe for SRO. These values are very close to the experimental values that are directly determined by the saturation field in IP loops, where the anisotropy fields are around 7.8 kOe for BFO, 8.3 kOe for PTO, 30 kOe for STO, and 37 kOe for SRO. The coincidence of magnetic anisotropy field confirms the variation of anisotropy constant used in simulating these loops. These results also imply that higher strain state of the CFO mesocrystal drives the alteration of *k*_12_, which has been observed in those cases of off-stoichiometric CFO due to the cation site-exchange of Co and Fe ions^26^,^27^. Therefore, there should be a strong correlation between strain state and the cation redistribution in the CFO mesocrystal. This speculation is then verified by performing X-ray magnetic circular dichroism (XMCD) measurements, which is a useful technique to understand the charge, site symmetry and origin of magnetic moments in atomic scale.

**Figure 5:**
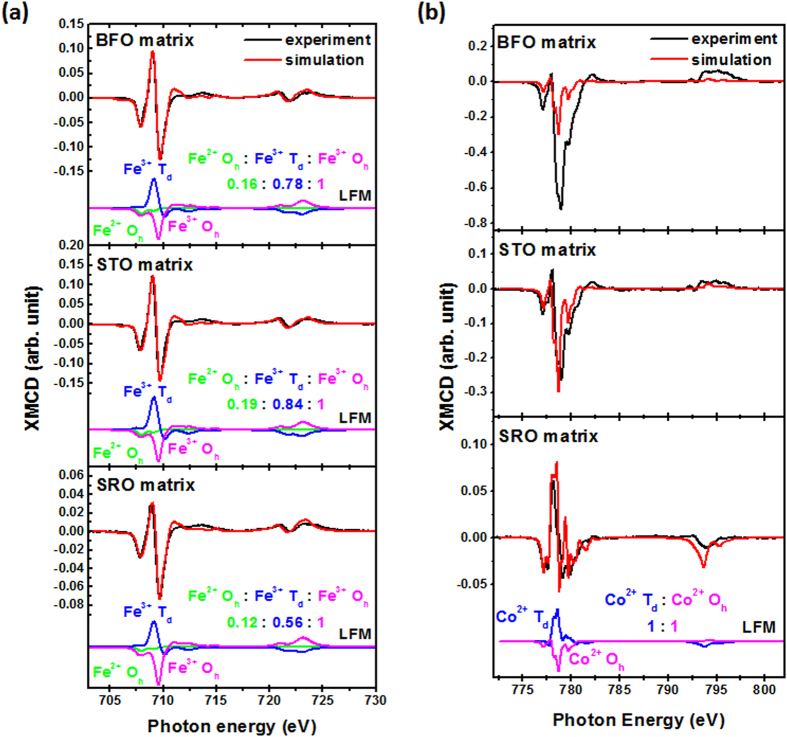
(**a**) Co and (**b**) Fe *L*_*3,2*_-edge XMCD spectra extracted from photo-helicity of incident X-rays parallel (*μ*^+^) and anti-parallel (*μ*^−^) to the direction of magnetization for the CFO mesocrystal in BFO, STO, and SRO matrices. By simulating the experimental curves using LFM model, the site ratio of tetrahedral site and octahedral site for Co and Fe cations can also be obtained, which shows an obvious redistribution between each cation and site that is highly correlated to the stress-mediated structural coupling.

Three samples selected for XMCD measurement are the CFO mesocrystals with BFO, STO, and SRO matrices, which represent the strain-free, medium strain, and highly strain states, respectively. By applying the site simulation based on the ligand field model (LFM), XMCD spectra at Fe *L*_2,3_ edges for these three samples (Fig. 5(a)) exhibit two negative peaks at 707.8 and 709.01 eV, and one positive peak at 709.6 eV, which are corresponding to the Fe^2+^ and Fe^3+^ cations at the octahedral (O_h_) sites, and Fe^3+^ cations at the tetrahedral (T_d_) sites^28^,^29^,^30^,^31^. In addition, the sample with SRO matrix exhibits a decreased peak intensity in Fe^3+^ T_d_ sites and an increased peak intensity in Fe^3+^ O_h_ sites compared to the other two samples, revealing that more Fe^3+^ cations prefer to occupy the O_h_ sites in this case. On the other hand, XMCD spectra measured at Co *L*_2,3_ edges (Fig. 5(b)) show a significant discrepancy among these three samples. Although, Co XMCD measured from the samples with BFO and STO matrices show the same line-shape and consistent with the reference XMCD of Co^2+^ at O_h_ sites^32^,^33^, the case of BFO matrix possesses a much larger XMCD signal comparing to the other two. Chen *et al.* proposed that this dramatic enhancement of magnetic moments in the case of BFO matrix comes from a strong magnetic coupling between the mesocrystal and the matrix^32^. The simulation of XMCD spectrum measured from the sample with SRO matrix reveals a mixture of Co^2+^ cations at the T_d_ and O_h_ sites^28^,^29^. The existence of Fe^2+^ O_h_ sites and Co^2+^ T_d_ sites suggests a site-exchange occurred in these systems because strain effect or oxygen vacancies can easily break the symmetry and redistribute cations and charges^28^,^29^,^30^,^31^. Based on the results of site simulation, the ratio of Fe^3+^ T_d_ sites and O_h_ sites can be obtained as ~0.78 for nearly strain-free CFO, ~0.84 for medially strained CFO, and ~0.56 for highly strained CFO. The Co^2+^ cations solely occupy at the O_h_ sites for nearly strain-free and medially strained cases, which remain similar electronic structures close to the CFO references. The highly strained CFO has a ratio of Co^2+^ T_d_ sites and O_h_ site close to one.

## Discussion

**Figure 6:**
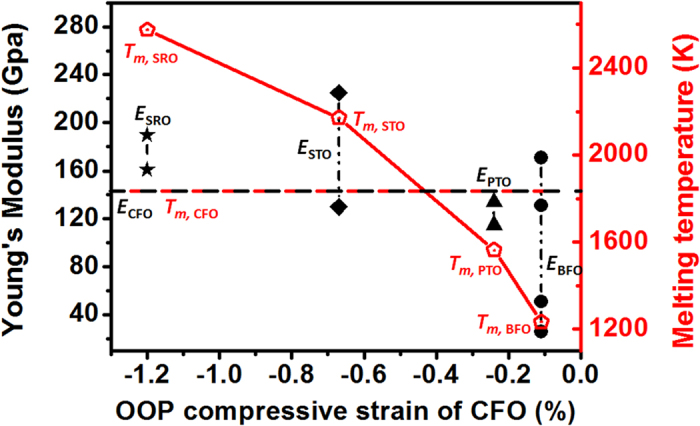
The relationship between Young’s modulus (*E*) or melting temperature (*T*_*m*_) of the perovskite matrices according to Table 1, and the OOP compressive strain of the mesocrystal CFO. The black and red dash line represents the Young’s modulus (142 GPa) and melting temperature (1840 K) of CFO, respectively. These results imply that the matrices with higher bonding strength can cause larger OOP compressive strain of the mesocrystal CFO.

By overviewing the structural and magnetic information, the strain-mediated magnetic properties of the CFO mesocrystal have been confirmed to be very different in these matrices. A tantalizing question is: can we discover any tendency among these matrices rather than the lattice mismatch between the mesocrystals and the matrices? In order to answer this question, we first start from the origin of strain in the cases of thin films or multilayers. It is well known that the substrates can be treated as semi-infinite matrices and rigid bodies comparing to the deposited thin films/multilayers. Therefore, the strain experienced by the film materials could be determined by the lattice mismatch between the films and the substrates. Conversely, in the self-assembled nanocomposites, the mesocrystal and matrix have comparable volume (the molar ratio of CFO in each sample is around 0.33) and both may possibly affect each other, so that the structural coupling cannot only be determined by the lattice mismatch. For example, there is only very little difference in lattice parameters between pseudo-cubic BFO and SRO, where *a*_BFO_ is around 3.96 Å and *a*_SRO_ is around 3.93 Å. However, the CFO mesocrystal is almost strain-relaxed in BFO matrix and has a large OOP compressive strain (~−1.2%) in SRO matrix. This implies that the mechanism to cause the OOP compressive strain of the CFO mesocrystal depends on the competition of the bonding strength of these materials. From the basic physics behind the rigid solids, the bonding strength can be correlated to some parameters such as melting point and stiffness. The corresponding information of each material is listed in Table 1. We found out that the melting point (*T*_*m*_) and Young’s modulus (*E*) of matrix materials show positive correlations with the strain state of the CFO mesocrystal (Fig. 6). It clearly maps out that the OOP compressive strain and magnetic anisotropic field of the CFO mesocrystal increases with the melting point and Young’s modulus of perovskite matrices. Although the Young’s modulus of each perovskite matrix obtained from different references have a deviation, which may result from different experimental setups or film qualities, they show the same trend as the melting points. Generally, the matrix with larger bonding strength also implies that it has stronger atomic linkage and larger stiffness. Such ability can be even extended to the adjoining atoms of the CFO mesocrystal at interfaces and thus compressively deforms the CFO lattice. The evidences from the TEM results reported in several previous works have exhibited that the spinel mesocrystal shows a larger distortion and less dislocations at interfaces while connecting to the SRO matrix^14^, whereas it has smaller distortion and more dislocations at interfaces while connecting to the BFO matrix^10^. To tolerate the high strain at largely mismatched interfaces, defects such as cation site substitutions instead of dislocations are a good choice. Namely, our finding is that the surrounding matrices determine the strain states and defect types of the CFO mesocrystal. This is very fascinating because this kind of mesocrystals exhibits tunable functionalities by just modifying the neighboring matrix materials, which cannot be achieved in traditional ones obtained from chemical approaches.

In conclusion, the 2D CFO mesocrystal composed of a large amount of orientation-ordered nanocrystals has been obtained from an oxide-based nanocomposite system using PLD process. Unlike those mesocrystals embedded in organic/polymer matrices obtained from the chemical synthesis, the oxide matrix plays a decisive role to tailor the structure and functionalities of the mesocrystal. By taking the spinel-perovskite nanocomposites as model systems, the CFO mesocrystal shows a dramatic change of strain state and magnetic behaviors while varying the matrix materials. The strain variation of CFO with perovskite matrices not only changes the magnetic anisotropy, but also leads to the redistribution of cations. Such a variation in the properties of the CFO mesocrystal has been demonstrated to strongly correlate to degree of the structural coupling with the matrices. More interestingly, it can be directly associated with the bonding strength of perovskite matrices, which are reflected in the melting point or stiffness. This study paves an elegant way for tuning the strain-mediated functionalities of this mesocrystal, only by selecting the proper matrix material referring to its intrinsic material properties such as melting point or stiffness, which creates a new degree of freedom to engineer such mesocrystal systems.

## Methods

All CFO mesocrystals were fabricated on (001) oriented SrTiO_3_ (STO) substrates by pulsed laser deposition (PLD) with a KrF (λ = 248 nm) excimer laser. The samples of CFO mesocrystals in BFO, PTO, and STO matrices are prepared using the mixed targets with the nominal molar ratio (*m*_CFO_/(*m*_CFO_ + *m*_Matrix_)) of 1/3. The sample of CFO mesocrystal in SRO matrix is fabricated by dual-target process that is following our previous work^14^. The molar ratio is determined according to the deposition rate of each SRO and CFO single target (0.043 Å/pulse for SRO and 0.02 Å/pulse for CFO). A combination of AFM and TEM has been used to verify the percentage of CFO to ensure that all the samples contains the same amount of CFO (not shown here). In addition, the samples of CFO mesocrystals in BFO and PTO matrices were deposited at the substrate temperature of 650 °C and in a dynamic oxygen pressure of 100 mTorr because these two matrices are easier to decompose at the temperature higher than 700 °C. Other samples were deposited at substrate temperature of 750 °C and at the same oxygen pressure. After the growth, all samples were cooled to room temperature in oxygen pressure ~1 atm. High-resolution symmetry and asymmetry X-ray diffraction techniques were taken to verify the epitaxial relationship between the CFO mesocrystals and the perovskite matrices at beamline BL17B in the National Synchrotron Radiation Research Center (NSRRC). The micro-Raman scattering was measured using a LabRam Jobin-Yvon spectrometer equipped with the liquid nitrogen-cooled CCD and 532 nm excitation. The mode frequencies are fitted with Lorentzian functions after subtracting the signals from substrates. The magnetism of all samples was studied using superconducting quantum interference device magnetometry measurements (SQUID). The electronic structure and origin of magnetism of CFO were studied using soft X-ray absorption spectra (XAS) and X-ray magnetic circular dichroism (XMCD) at the Dragon beamline in NSRRC.

## Additional Information

**How to cite this article**: Liu, H.-J. *et al.* Tuning the functionalities of a mesocrystal via structural coupling. *Sci. Rep.* **5**, 12073; doi: 10.1038/srep12073 (2015).

## Figures and Tables

**Table 1:** The information about melting point (*T*_*m*_) and Young’s modulus (*E*) of the matrices and CoFe_2_O_4_, and the respective OOP strain (*ε*_*OOP*_ ), IP strain (*ε*_*IP*_), OOP coercievity (*H*_*C,OOP*_), the magnetic anisotropy field obtained according to Eq. 1 (*K*_*stress,cal*._) and experiments (*K*_*stress,exp*._) of mesocrystal CFO in these matrices.

	**BiFeO**_**3**_	**PbTiO**_**3**_	**SrTiO**_**3**_	**SrRuO**_**3**_	**CoFe**_**2**_**O**_**4**_
*T*_*m*_	1233^34^	1563^35^	2170^36^	2575^36^	1840^37^
*E*	26 ~ 51^38^,^39^	115^40^	130^41^	161^36^	142^12^
	131 ~ 171^42^	134^42^	225^43^	190^41^	
*ε*_*OOP*_	−0.11%	−0.24%	−0.67%	−1.2%	
*ε*_*IP*_	0.05%	0.1%	0.41%	1%	
*H*_*C,OOP*_	2460	2330	6240	8640	
*K*_*stress,cal*_	7.5 kOe	8.4 kOe	29.9 kOe	37.3 kOe	
*K*_*stress,exp*_	7.8 kOe	8.3 kOe	30 kOe	37 kOe	
